# Nephrotoxin-Induced Renal Cell Injury Involving Biochemical Alterations and Its Prevention With Antioxidant

**DOI:** 10.4021/jocmr833w

**Published:** 2012-03-23

**Authors:** Andrew I. Fishman, Bobby Alexander, Majid Eshghi, Muhammad Choudhury, Sensuke Konno

**Affiliations:** a Department of Urology, New York Medical College, Valhalla, New York, USA

## Abstract

**Background:**

Although nephrotoxic agents or nephrotoxins are known to induce acute renal cell injury, their cytotoxic action is not fully elucidated. It is thus crucial to explore such a cytotoxic mechanism and the increasing volume of reports indicated a significant involvement of oxidative stress. To test this possibility, we investigated if a nephrotoxin would exert oxidative stress, leading to renal cell injury accompanied by certain biochemical alterations. We also examined if specific antioxidant might help prevent such oxidative cell injury. These studies may then help establish a prophylactic or preventive modality for renal cell injury induced by nephrotoxins.

**Methods:**

As glycerol has been commonly used for studying acute renal failure in animals, whether it would induce cellular injury was tested in renal proximal tubular OK cells *in vitro*. Cells were exposed to the varying concentrations of glycerol and cell number/viability was determined in 24 hours. Severity of oxidative stress was assessed by lipid peroxidation assay. Possible effects of glycerol on biochemical parameters were also examined on glyoxalase I activity and heat shock protein 90 using spectrophotometric (enzymatic) assay and Western blot analysis.

**Results:**

Glycerol (2.5%) was highly cytotoxic to OK cells, inducing 95% cell death in 24 hours. Lipid peroxidation assay indicated that nearly 3-fold greater oxidative stress was exerted by this glycerol. Concurrently, glyoxalase I activity was drastically lost by 75% and heat shock protein 90 was partially degraded following glycerol exposure. However, N-acetylcysteine, a potent glutathione-based antioxidant, was capable of almost completely preventing the glycerol-mediated adverse outcomes, such as cell death, glyoxalase I inactivation, and heat shock protein 90 degradation.

**Conclusions:**

Glycerol is cytotoxic, capable of inducing specific biochemical alterations such as inactivation of glyoxalase I and degradation of heat shock protein 90, which may reflect a breakdown of the cellular detoxification and defense systems, leading ultimately to OK cell death. Nevertheless, as N-acetylcysteine can provide full cytoprotection against such glycerol toxicity, it could be considered a prophylactic modality for nephrotoxin-induced oxidative renal cell injury and death.

**Keywords:**

Glycerol; Glyoxalase I; Heat shock protein; N-acetylcysteine; Renal cell injury

## Introduction

Various nephrotoxic agents, such as HgCl_2_, glycerol, cisplatin, gentamicin etc., have been known to exert cytotoxic effects on renal tubular cells [1, 2], inducing acute renal cell injury. However, as their cytotoxic action yet remains elusive, the preventive or therapeutic modalities for renal cell injury have not been established. Nevertheless, the accumulating data suggest that the involvement of oxidative stress (generation of oxygen free radicals) could be crucial for such renal cell injury induced by nephrotoxins [3, 4]. In addition, we have recently reported that oxidative stress would play a primary role in renal cell injury with certain nephrotoxins [5]. We were thus encouraged to further expand our study of nephrotoxin-mediated oxidative stress, focusing on biochemical parameters in the different renal cell line.

One of such biochemical parameters is glyoxalase I (Gly-I), a vital enzyme involved in the cellular detoxification process [6], playing a critical role in detoxifying and scavenging cytotoxic agents and metabolites (including free radicals) [7, 8]. Since activation of Gly-I inevitably requires reduced glutathione (GSH) as a cofactor [7], it is also categorized in a family of GSH-dependent enzymes. It is then plausible that Gly-I could be somewhat involved in detoxification of nephrotoxins.

A family of heat shock proteins (Hsps) is another interesting biochemical parameter, consisting of several species [9]. As they are functionally known as stress-response proteins, immediately responding to a variety of stresses exerted on the cells, they have been considered to play an important role in the cellular defense mechanism [10]. Among those Hsps, we are particularly interested in heat shock protein 90 (Hsp90) because it is primarily localized in the distal tubules and collecting ducts in the kidney [11] and would serve to protect renal cells from stress-related assaults [12]. It is thus possible that Hsp90 may also help protect renal cells from certain nephrotoxins.

We proposed that nephrotoxins would exert oxidative stress on renal cells as their primary cytotoxic action, triggering a cascade of biochemical events and leading ultimately to cell death. Accordingly, glycerol (GLC), a nephrotoxin capable of inducing renal cell injury, was chosen for studying its cytotoxic action on renal proximal tubular OK cells [13] *in vitro* (instead of LLC-PK_1_ cells used in our previous study). We first examined if GLC would actually exert oxidative stress on OK cells, and possible effects of GLC (through oxidative stress) were then examined on two biochemical parameters, Gly-I and Hsp90. We also explored if certain antioxidants (e.g. vitamins C/E and GSH) might provide cytoprotection against GLC-mediated oxidative assault. Detailed studies and notable findings are described and discussed herein.

## Materials and Methods

### Cell culture

The renal proximal tubular OK cells [13] were maintained in RPMI-1640 medium supplemented with 10% fetal bovine serum, penicillin (100 units/ml), and streptomycin (100 μg/ml). For experiments, OK cells were seeded at 2 × 10^5^ cells/ml in 6-well plates or T-75 flasks and cultured with varying concentrations of glycerol (GLC). Cell number/viability was determined at specified times by the trypan blue exclusion method.

### Lipid peroxidation (LPO) assay

Severity of oxidative stress on the cells was assessed by LPO assay measuring the amount of malondialdehyde (MDA) formed, which was indicative of oxidative damage in the plasma membrane [14]. The detailed procedures were described in the vendor’s protocol (Calbiochem, La Jolla, CA), and the amount of MDA formed was expressed by μM determined from the MDA standards.

### Glyoxalase I (Gly-I) assay

Gly-I activity was measured following the method of Ranganathan and Tew [15]. After preparation of the reaction mixture (200 mM imidazole HCl, pH 7.0, 16 mM MgSO_4_, 7.9 mM methylglyoxal, 1 mM GSH), the reaction was started by the addition of cell lysates (40 μg). Due to a production of S-D-lactoylglutathione (E_240_ = 3.37 mM^-1^· cm^-1^), the increase in absorbance at 240 nm was measured with times on a spectrophotometer. Gly-I activity was then expressed by units/mg protein where one unit was defined to catalyze the formation of one μmol of S-D-lactoylglutathione per min.

### Western blot analysis

The procedures essentially followed the protocol described previously [16]. Briefly, an equal amount of cell lysates (7 μg) obtained from control and agent-treated cells was subjected to 10% SDS-polyacrylamide gel electrophoresis and transferred to a nitrocellulose membrane. The blot (membrane) was incubated with the primary antibody against Hsp90 (anti-Hsp90), followed by an incubation with the secondary antibody conjugate. The immunoreactive protein bands were then detected by chemiluminescence following the manufacturer’s protocol (Kirkegaard and Perry Laboratories, Gaithersberg, MD).

### Statistical analysis

All data were presented as mean ± SD (standard deviation), and statistical differences between groups were assessed with either one-way analysis of variance (ANOVA) or the unpaired Student’s *t* test. Values of P < 0.05 were considered to indicate statistical significance.

## Results

### Effects of glycerol (GLC) on OK cell proliferation

**Figure 1:**
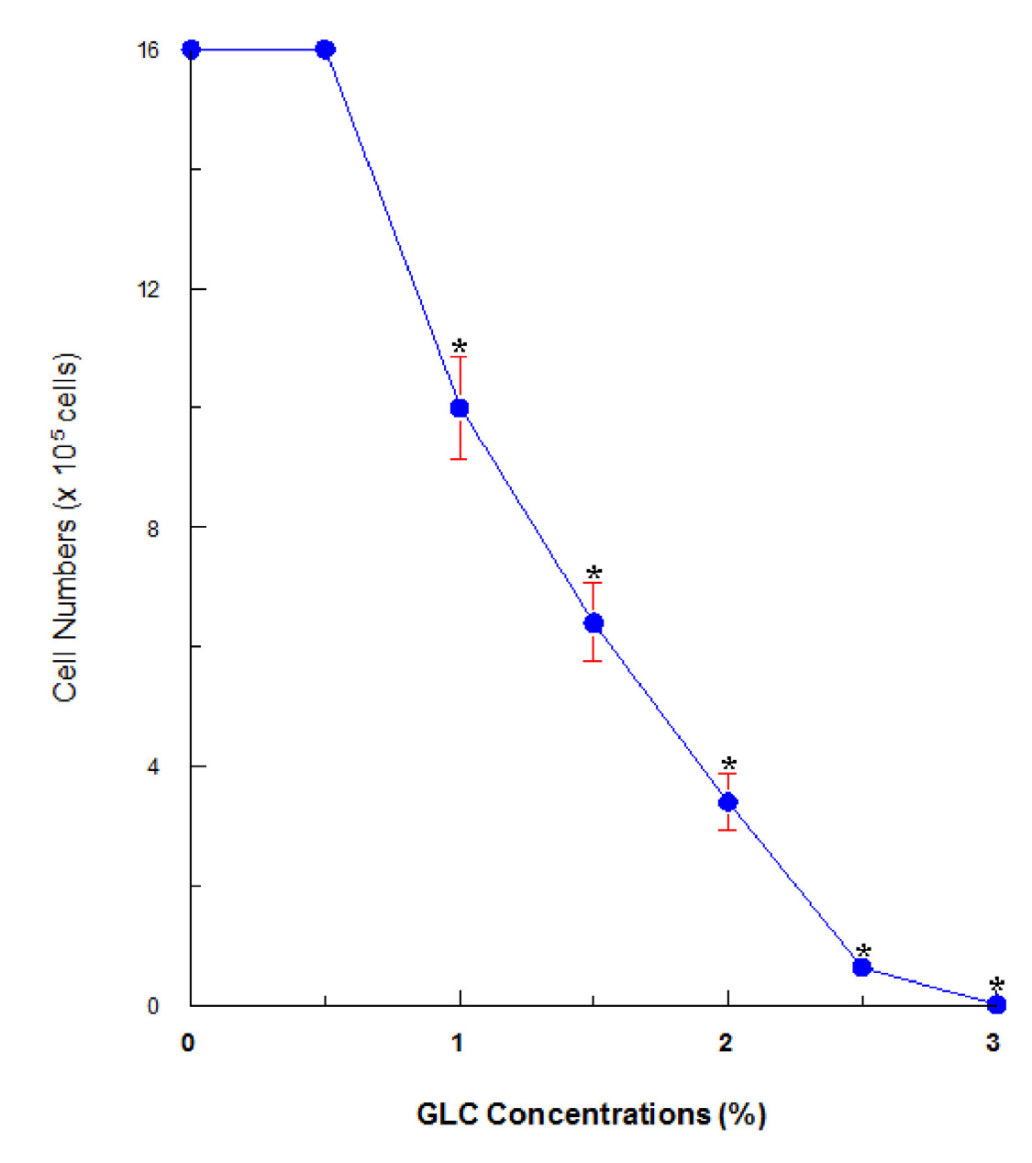
Dose-dependent effects of glycerol (GLC) on OK cell growth. Cells were cultured with varying concentrations (0, 0.5, 1, 1.5, 2, 2.5, or 3%) of GLC for 24 hours, and cell numbers were determined as mean ± SD (standard deviation) from three separate experiments (*P < 0.03).

OK cells were cultured with varying concentrations (0 - 3%) of GLC and cell growth/viability was determined in 24 hours. GLC was capable of inducing 38%, 60%, 79%, 95%, and 100% growth reduction with 1%, 1.5%, 2%, 2.5%, and 3% GLC, respectively (Fig. 1). Although no apparent cell death was observed up to 2% GLC, a 95% growth reduction attained with 2.5% GLC was largely attributed to severe cell death. We then used this 2.5% GLC as the most effective concentration in the rest of our study.

### Oxidative stress exerted by GLC

To understand how GLC could induce such severe cell death, the possible involvement of oxidative stress was next examined as it had been previously addressed [3, 4]. LPO assay was performed to assess severity of oxidative stress as described in Materials and Methods. The MDA level in the cells exposed to GLC for 3 hours was ~3-fold greater than that in controls, indicating severe plasma membrane damage through oxidative stress (Table 1). Thus, GLC appears to markedly exert oxidative stress on the cells, eventually leading to renal cell death.

**Table 1:** Effects of GLC on LPO Levels Measured by MDA Formed

Conditions	MDA Formed (μM)^Δ^ at 3 Hours	
Control	0.87 ± 0.09	(1)*
+ GLC (2.5%)	2.71 ± 0.22	(3.1)*

^Δ^ Mean ± SD (standard deviation) of three separate experiments. * Values in parentheses are arbitrary numbers relative to control’s (1 = 0.87 μM).

### Cytoprotection provided with N-acetylcysteine against GLC

**Figure 2:**
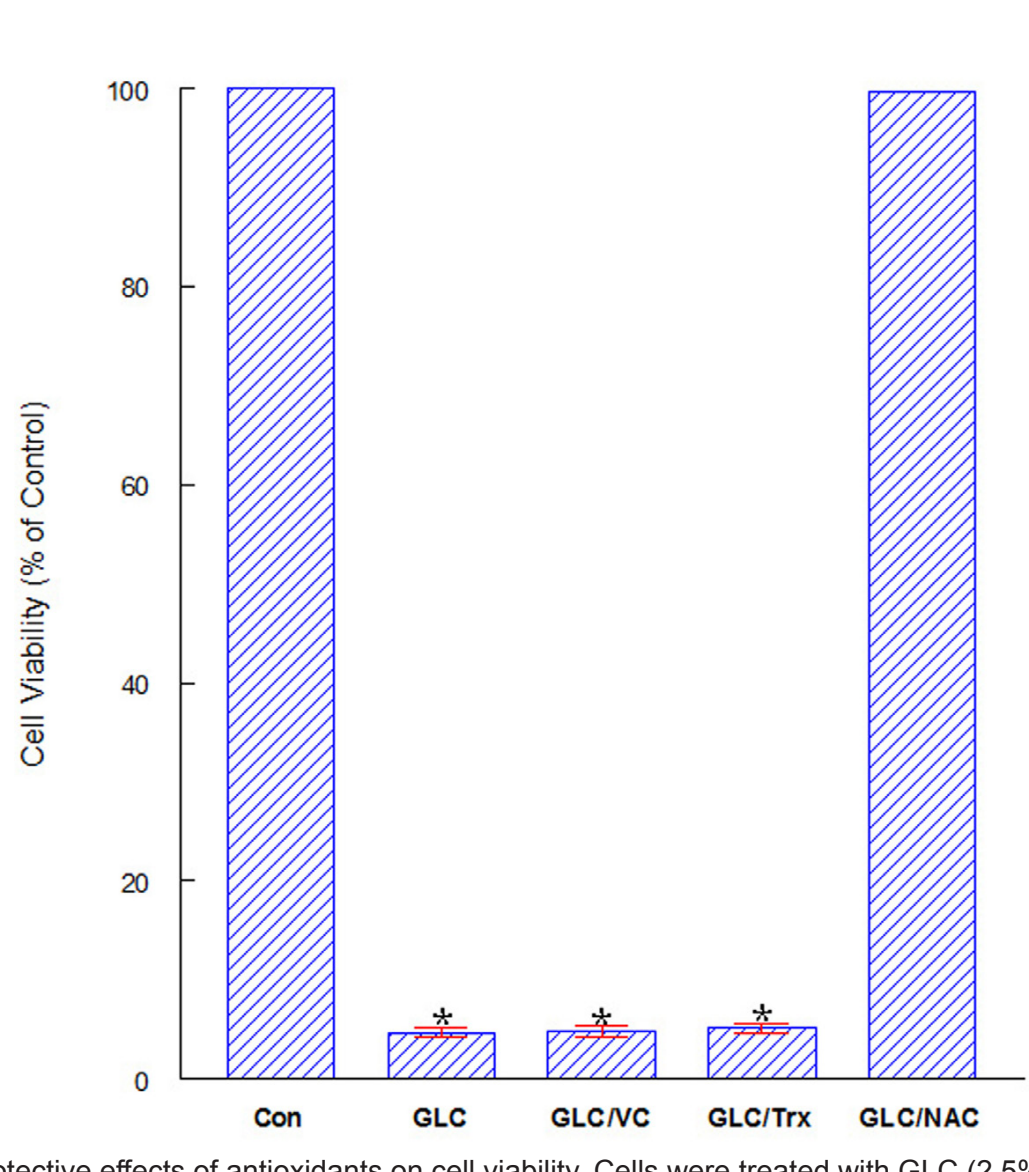
Protective effects of antioxidants on cell viability. Cells were treated with GLC (2.5%) alone or in combination with VC (200 μM), Trx (300 μM), or NAC (500 μM) and cell viability was assessed in 24 hours. All data are mean ± SD from three separate experiments (*P < 0.01 compared to controls).

As antioxidants are known to be effective against oxidative stress, we examined if certain antioxidants such as vitamin C (VC), trolox (Trx; a cell-permeable, water-soluble derivative of vitamin E) [17], or N-acetylcysteine (NAC; a cell-permeable precursor for GSH) [18] could protect OK cells from GLC-mediated oxidative stress. GLC (2.5%) alone induced ~95% cell death in 24 hours but NAC (500 μM) prevented such severe cell death, maintaining cell viability at nearly 100% (Fig. 2). No such protective effects were yet seen with VC (200 μM) or Trx (300 μM). Thus, NAC is capable of providing cytoprotection against GLC oxidative assault.

### Possible role of glyoxalase I (Gly-I) in NAC-provided cytoprotection

**Figure 3:**
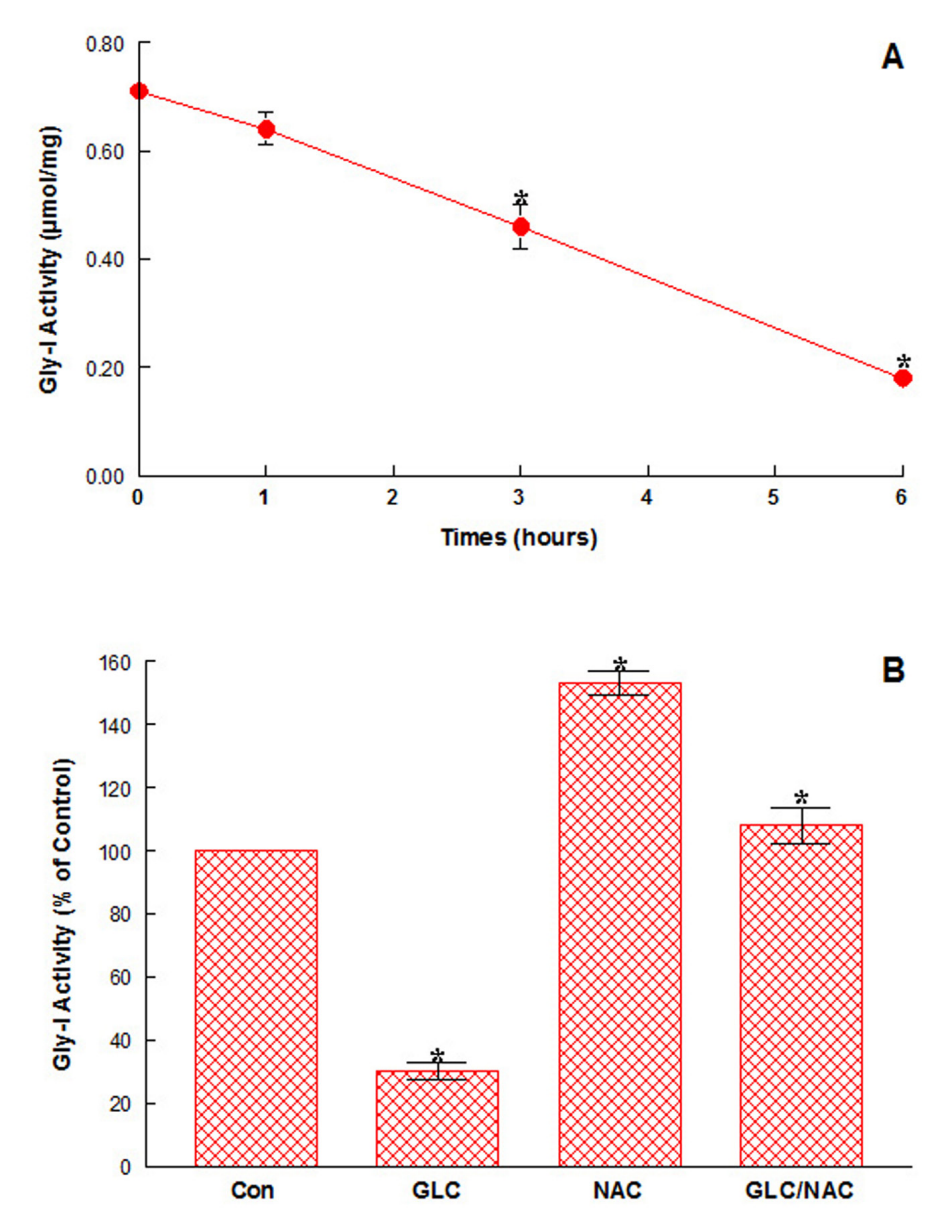
A: Time-dependent effects of GLC on Gly-I activity. OK cells were exposed to GLC (2.5%) for 1, 3, or 6 hours, and Gly-I activity was measured and expressed by (mol/mg, as described in Materials and Methods. All data are mean ± SD from three separate experiments (*P < 0.03). B: Effects of NAC on GLC-induced Gly-I inactivation. Cells were exposed to GLC (2.5%), NAC (500 μM), or GLC/NAC combination for 6 hours, and Gly-I activity was determined and expressed by the % relative to control’s (100% Gly-I activity = 0.71 μmol/mg). The data are mean ± SD from three independent experiments (*P < 0.05 compared to controls).

The finding that only NAC was effective against oxidative assault led us to assume that NAC might not only scavenge free radicals but also act as some biochemical factor. In fact, NAC is known as an essential co-factor required for activation of glyoxalase I (Gly-I), which plays a major role in cellular detoxification [6]. The possible involvement of Gly-I in NAC cytoprotection was then examined. Such study showed that Gly-I activity was significantly lost by 35% and 75% with 3- and 6-hour GLC exposure, respectively (Fig. 3A). In contrast, NAC (500 μM) was capable of not only enhancing basal Gly-I activity (~50% greater than controls) but also completely preventing Gly-I inactivation with 6-hour GLC exposure (Fig. 3B). With such a direct impact of NAC on Gly-I, it is plausible that NAC-provided cytoprotection could be in part attributed to Gly-I mediated detoxification of GLC.

### Effects of GLC on heat shock protein 90 (Hsp90)

**Figure 4:**
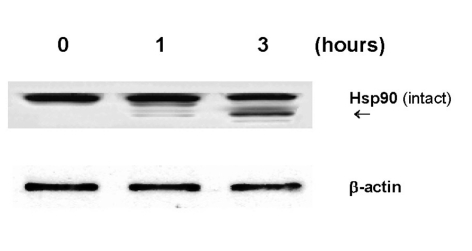
Effect of GLC on Hsp90. Cells exposed to GLC (2.5%) for indicated times were analyzed for expression of Hsp90 on Western blots. The degraded product (~85 kDa) of Hsp90 is indicated by an arrow, and β-actin is used as a protein loading control.

We were also interested in another biochemical parameter known as Hsp90, because it has been shown to play a protective role against various types of cytotoxic insults [10]. OK cells exposed to GLC (2.5%) for 1 or 3 hours were analyzed for the status of Hsp90 on Western blots. Partial degradation of Hsp90 was detected at 1 hour and became clearly evident at 3 hours (Fig. 4), exhibiting a lower degraded protein band (~85 kDa). This finding further indicates a possible breakdown of the cellular defense system (by GLC), which could feasibly lead to cell injury and even cell death.

## Discussion

As our previous study [5] suggested a primary role of oxidative stress in renal cell injury induced by nephrotoxins, we further investigated possible alterations in specific biochemical events induced by a nephrotoxin, glycerol (GLC), in renal tubular OK cells *in vitro*. GLC (2.5%) demonstrated to be highly cytotoxic, inducing severe cell death (95%) in 24 hours. Such cytotoxicity appeared to be primarily due to oxidative stress through GLC, indicated by LPO assay (Table 1). It is thus conceivable that such oxidative stress may trigger a cascade of various biochemical events, leading ultimately to cell death.

We next examined if certain antioxidant(s) might counteract with GLC-mediated oxidative stress. Interestingly, only NAC was found to protect OK cells from GLC oxidative assault (Fig. 2), implying the possible involvement of specific NAC-activated enzyme such as glyoxalase I (Gly-I) [6]. Our study then showed that a 75% loss in Gly-I activity after 6-hour GLC exposure (Fig. 3A) was completely reversed or prevented with NAC (Fig. 3B) and cell viability also remained at nearly 100% (Fig. 2). Thus, these results suggest that Gly-I may somewhat detoxify/diminish GLC cytotoxicity, contributing to NAC-provided cytoprotection.

It is crucial but remains uncertain how GLC would inactivate Gly-I. Yet, we assume that GLC-induced Gly-I inactivation may likely result from an insufficient availability of cellular GSH (synthesized in renal cells) because activation of Gly-I essentially requires GSH (or NAC used in this study) [7]. It is possible that GLC by itself or GLC-mediated oxidative stress may interfere with de novo synthesis of GSH, depriving cellular GSH. For example, γ-glutamylcysteine synthetase [18], a key enzyme involved in GSH synthesis, or other enzymes could be primarily targeted by GLC or free radicals. Further studies are thus required.

Another interesting biochemical alteration through GLC was partial degradation of Hsp90 (Fig. 4). As Hsp90 is shown to play a defensive role, its degradation could result in a weakening or destabilization of the cellular defense system, ultimately leading to renal cell death. It is yet of interest to further explore how Hsp90 would be degraded by GLC. As protein degradation is generally known to be carried out by protease(s), we assume that proteasome, a multicatalytic protease, might be involved in Hsp90 degradation because it has been shown to play a major role in degradation of intracellular proteins including Hsps [19]. Such study is currently underway in our laboratory.

In summary, the cytotoxic action of GLC primarily involves oxidative stress, causing adverse biochemical alterations such as inactivation of Gly-I and partial degradation of Hsp90. This indicates a collapse of the cellular detoxification and defense systems, presumably leading to renal cell death. However, NAC-provided cytoprotection against GLC, restoring Gly-I activity and protecting Hsp90 integrity, may have clinical implications in preventing renal cell injury/death induced by nephrotoxins.
